# The Quality of Health Apps and Their Potential to Promote Behavior Change in Patients With a Chronic Condition or Multimorbidity: Systematic Search in App Store and Google Play

**DOI:** 10.2196/33168

**Published:** 2022-02-04

**Authors:** Alessio Bricca, Alessandro Pellegrini, Graziella Zangger, Jonas Ahler, Madalina Jäger, Søren T Skou

**Affiliations:** 1 Research Unit for Musculoskeletal Function and Physiotherapy Department of Sports Science and Clinical Biomechanics University of Southern Denmark Odense Denmark; 2 The Research Unit PROgrez Department of Physiotherapy and Occupational Therapy Næstved-Slagelse-Ringsted Hospitals Slagelse Denmark

**Keywords:** app, self-management, behavior change, multimorbidity, chronic conditions, health apps, mHealth, mobile health, mobile phone

## Abstract

**Background:**

Mobile apps offer an opportunity to improve the lifestyle of patients with chronic conditions or multimorbidity. However, for apps to be recommended in clinical practice, their quality and potential for promoting behavior change must be considered.

**Objective:**

We aimed to investigate the quality of health apps for patients with a chronic condition or multimorbidity (defined as 2 or more chronic conditions) and their potential for promoting behavior change.

**Methods:**

We followed the Cochrane Handbook guidelines to conduct and report this study. A systematic search of apps available in English or Danish on App Store (Apple Inc) and Google Play (Google LLC) for patients with 1 or more of the following common and disabling conditions was conducted: osteoarthritis, heart conditions (heart failure and ischemic heart disease), hypertension, type 2 diabetes mellitus, depression, and chronic obstructive pulmonary disease. For the search strategy, keywords related to these conditions were combined. One author screened the titles and content of the identified apps. Subsequently, 3 authors independently downloaded the apps onto a smartphone and assessed the quality of the apps and their potential for promoting behavior change by using the Mobile App Rating Scale (MARS; number of items: 23; score: range 0-5 [higher is better]) and the App Behavior Change Scale (ABACUS; number of items: 21; score: range 0-21 [higher is better]), respectively. We included the five highest-rated apps and the five most downloaded apps but only assessed free content for their quality and potential for promoting behavior change.

**Results:**

We screened 453 apps and ultimately included 60. Of the 60 apps, 35 (58%) were available in both App Store and Google Play. The overall average quality score of the apps was 3.48 (SD 0.28) on the MARS, and their overall average score for their potential to promote behavior change was 8.07 (SD 2.30) on the ABACUS. Apps for depression and apps for patients with multimorbidity tended to have higher overall MARS and ABACUS scores, respectively. The most common app features for supporting behavior change were the self-monitoring of physiological parameters (eg, blood pressure monitoring; apps: 38/60, 63%), weight and diet (apps: 25/60, 42%), or physical activity (apps: 22/60, 37%) and stress management (apps: 22/60, 37%). Only 8 out of the 60 apps (13%) were completely free.

**Conclusions:**

Apps for patients with a chronic condition or multimorbidity appear to be of acceptable quality but have low to moderate potential for promoting behavior change. Our results provide a useful overview for patients and clinicians who would like to use apps for managing chronic conditions and indicate the need to improve health apps in terms of their quality and potential for promoting behavior change.

## Introduction

Osteoarthritis, hypertension, type 2 diabetes, depression, heart conditions, and chronic obstructive pulmonary disease are among the leading causes of global disability [1]. These conditions affect millions of people worldwide and are commonly co-occurring (ie, multimorbidity) [2]. Patients with chronic conditions have poorer physical and psychosocial health than those of people without such conditions, and the higher the number of co-occurring conditions, the greater the impact on the individual and society [3,4]. Importantly, these conditions can be prevented and managed by a healthy lifestyle, highlighting the importance of investigating this population [5].

A healthy lifestyle, which includes physical activity and a healthy diet, is associated with up to a 6.3-year longer lifespan for men and a 7.6-year longer lifespan for women with 1 or more chronic conditions [6]. Different care models and interventions have been designed and implemented for people with multiple chronic conditions [7]. Although there is a paucity of information about the effects of these interventions, physical activity appears safe and beneficial for people with 1 or more chronic conditions [8,9]. The use of mobile apps to improve lifestyle has increasingly gained attention, particularly among patients with chronic conditions at any stage of their lives [10-14]. Apps may offer an opportunity to improve the lifestyles of patients with chronic conditions through, for example, self-monitoring and behavior change by providing access to personalized support and motivation anytime [15,16]. However, although apps are widely used (in 2019, more than 204 billion apps were downloaded) [17], their quality (eg, engagement and functionality), content, and potential for promoting behavior change are unclear [18]. Therefore, this study aimed to provide an overview of available health apps and their quality, content, and potential to promote behavior change in patients living with a chronic condition or multimorbidity.

## Methods

This systematic search of health apps was guided by the recommendations for performing systematic reviews in the Cochrane Handbook [19], and the protocol was made available prior to the app screening phase on Open Science Framework [20].

### Eligibility Criteria

We included apps that targeted lifestyle behaviors, such as physical activity and diet, and were directed at patients with 1 or more of the following conditions (multimorbidity): osteoarthritis of the knee or hip, heart conditions (heart failure and ischemic heart disease), hypertension, type 2 diabetes mellitus, chronic obstructive pulmonary disease, and depression. The rationale for focusing on these conditions was that they share a common risk factor (physical inactivity) and pathogenesis (systemic low-grade inflammation) and the fact that they are highly prevalent and can co-occur with each other. Therefore, the anti-inflammatory effects of lifestyle behaviors may improve the health of this population [21].

### Search Strategy and App Selection

We searched the Apple App Store (Apple Inc; iOS) and Google Play Store (Google LLC; Android) for Danish and English apps. Two authors (AB and AP) designed the search strategy (Table S1 in Multimedia Appendix 1), which was adapted from a prior systematic review [8,22]. One author (AP) performed the search in October 2020 and screened the titles and descriptions of the apps. Three authors (AP, GZ, and JA) independently downloaded the apps onto a smartphone. In pairs, they assessed the quality of the apps and their potential for promoting behavior change by using the Mobile App Rating Scale (MARS; number of items: 23) and the App Behavior Change Scale (ABACUS; number of items: 21), respectively. The five highest-rated apps and the five most downloaded apps were included. Quality and potential for promoting behavior change were only assessed for the free apps, given that the cost to purchase apps is a barrier to using mobile health apps [23]. Furthermore, apps were excluded if they did not target patients (eg, apps that targeted clinicians or organizations).

### Data Extraction and Outcomes

A complete overview of the data extraction process is available in the study protocol [20]. The following outcomes were assessed: app quality and the potential to promote behavior change.

App quality was assessed by using the MARS [24]. This validated and objective tool allows for the classification and assessment of the quality of apps. It is a 23-item scale that includes the following five categories: engagement, functionality, aesthetics, information quality, and subjective quality. Each item is assessed on a 5-point scale (1=inadequate; 2=poor; 3=acceptable; 4=good; 5=excellent).

The potential for behavior change was assessed by using the ABACUS [25]. This validated and objective tool includes 21 items that are grouped into the following four categories: knowledge and information, goals and planning, feedback and monitoring, and actions. The score for each item is dichotomous (yes or no), and an overall score (range 0-21) can be calculated. The higher the score, the higher the potential for promoting behavior change.

In addition, we extracted the characteristics of the apps, such as the number and type of self-monitoring tools (eg, a step count and BMI calculator), by using the 42matters website [26] for data that were not available in the iOS and Android stores. Mean scores for the MARS and ABACUS were calculated by averaging the ratings across all of the domains of the scales. The SDs were estimated accordingly.

### Synthesis of Results

We performed a narrative synthesis of the results and presented the results in a tabular and graphical format.

## Results

### App Selection and Characteristics

A total of 453 apps were identified, of which 150 were downloaded and screened for potential eligibility, and 60 were ultimately included (Figure S1 in Multimedia Appendix 1). Most of the included apps (35/60, 58%) were available for both iOS and Android. The apps were all available in English, and 25 out of the 60 apps (42%) were also available in other languages, including Danish, Arabic, and Chinese. The app size varied from 2.4 MB to 278 MB. Table S2 in Multimedia Appendix 1 presents a complete description of the apps.

### Quality of the Apps

The overall quality of the apps was acceptable (MARS score: mean 3.48, SD 0.28; range 0-5). However, apps for depression tended to have a higher overall MARS score (mean 3.89, SD 0.13), and apps for osteoarthritis tended to have a lower overall score (mean 3.0, SD 0.48) and lower scores for individual items of the MARS (Tables S3 and S4 in Multimedia Appendix 1).

### Apps’ Potential to Promote Behavior Change

**Figure 1:**
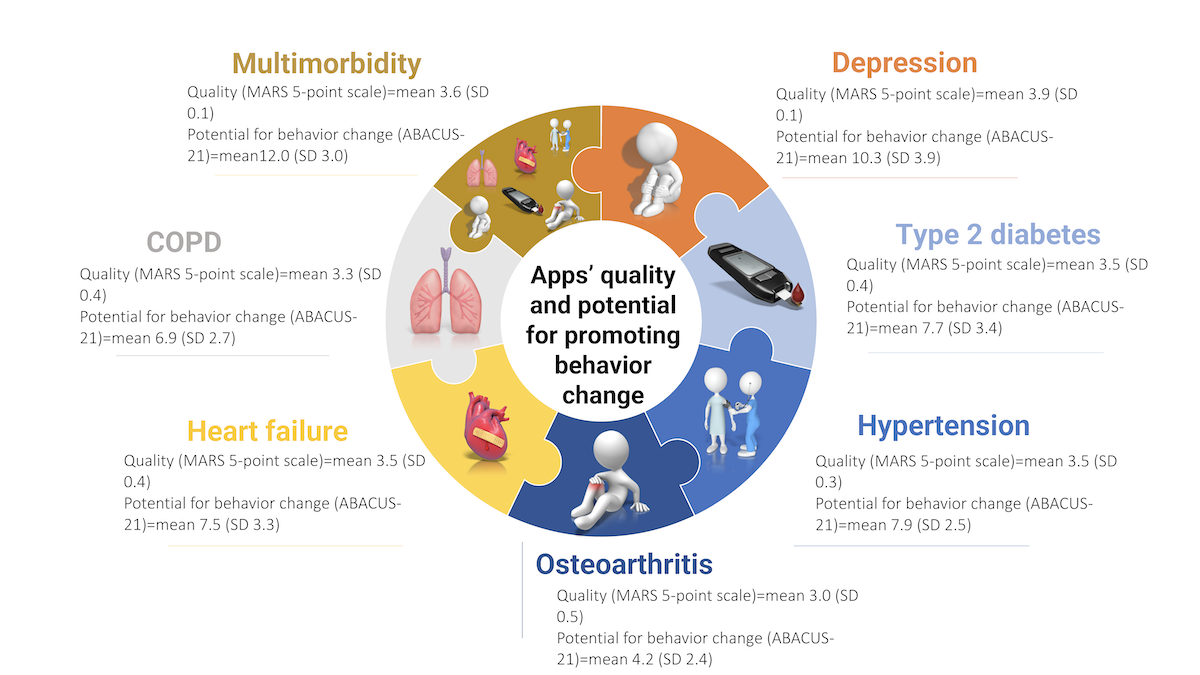
Summary of the findings for the quality of apps for osteoarthritis, hypertension, type 2 diabetes, depression, heart conditions, COPD, and multimorbidity and their potential for promoting behavior change. ABACUS: App Behavior Change Scale; COPD: chronic obstructive pulmonary disease; MARS: Mobile App Rating Scale.

The overall potential for behavior change was low to moderate (ABACUS score: mean 8.07, SD 2.30; range 0-21). Apps for patients with multimorbidity tended to have a higher overall ABACUS score (mean 12.0, SD 3.03), while apps for osteoarthritis tended to have the lowest overall scores (mean 4.22, SD 2.39) and the lowest scores for the individual categories of the ABACUS (Figure 1, Table S5 in Multimedia Appendix 1).

### Features of the Apps That Supported Behavior Change

The most common features presented in the apps that supported behavior change were the self-monitoring of physiological parameters (eg, blood pressure monitoring; apps: 38/60, 63%), weight and diet (apps: 25/60, 42%), or physical activity (apps: 22/60, 37%) and stress management (apps: 22/60, 37%). Only 8 out of the 60 apps (13%) were completely free.

## Discussion

### Principal Findings

To the best of our knowledge, this is the first study to assess the quality of health apps for patients with 1 or more chronic conditions and their potential for promoting behavior change. The assessed apps' quality is acceptable, but their potential for promoting behavior change in patients with osteoarthritis, hypertension, type 2 diabetes, depression, heart conditions, chronic obstructive pulmonary disease, or multimorbidity is low to moderate. This highlights the need for future studies to develop and evaluate apps with both high quality and high potential for promoting behavior change in patients with chronic conditions and multimorbidity.

The results of this study are comparable to the results of systematic reviews that investigated apps’ quality and potential for promoting behavior change in patients with a single chronic condition [27-29]. In these reviews, both low to moderate quality and low to moderate potential for promoting behavior change were found. Apps for multimorbidity tended to have higher quality and higher potential for promoting behavior change than those of apps for a single chronic condition despite the fact that research on multimorbidity is still in its infancy [30]. Future app studies should focus on improving quality and the potential for behavior change, especially among apps for conditions such as osteoarthritis, which had the lowest-quality apps [28,31]. Future studies should also test the effectiveness of apps via high-quality randomized controlled trials.

The features of the apps were similar across the chronic conditions, including multimorbidity, and focused mainly on the self-monitoring and tracking of physiological and behavioral parameters, such as medication intake, step count, and diet. In contrast, only a minority of apps (22/60, 37%) focused on psychosocial support, although mental and social health plays a major role in managing chronic conditions and multimorbidity [32-34]. This should be kept in mind when designing new apps.

Most of the top-rated and most downloaded apps for patients with a chronic condition or multimorbidity were not completely free (52/60, 87%). Notably, there were no free apps for depression. Nevertheless, the quality of apps for depression and their potential for behavior change were higher than those of apps for osteoarthritis, and 78% (7/9) of osteoarthritis apps were completely free. Although the development of apps has a cost, 1 in 2 smartphone users have never paid for an app [35], and the cost of apps is a barrier to using them [23]. This should be considered when designing new app-based interventions.

### Limitations

A possible limitation of this study is that we only assessed the free content of the apps. However, the potential for promoting behavior change appeared to be similar among free apps and apps with in-app purchases [27]. Furthermore, we only focused on English or Danish apps, meaning that our findings may not be generalizable to apps in other languages. We were also unable to identify apps targeting patients with ischemic heart disease and extract data on the characteristics of people who downloaded the apps (eg, age). This limited the generalizability of the findings related to apps for heart conditions to apps for patients with heart failure. This also limited our ability to conduct stratified subgroup assessments. Finally, the limited number of apps available for each condition prevented the meaningful comparison of the MARS and ABACUS subscales within and between conditions.

### Conclusions

Our results provide patients and clinicians with an overview of apps for managing 1 or more chronic conditions and indicate the need to improve the quality of apps and their potential for promoting behavior change, particularly among apps for patients with osteoarthritis.

## Appendix

Multimedia Appendix 1 Supplementary tables and figures.

## Abbreviations

ABACUS: App Behavior Change Scale
GLA:D: Good Life With osteoArthritis in Denmark
MARS: Mobile App Rating Scale
