# Supplementary material for: The Quality of Health Apps and Their Potential to Promote Behavior Change in Patients With a Chronic Condition or Multimorbidity: Systematic Search in App Store and Google Play
Source: JMIR Mhealth Uhealth. 2022 Feb 4;10(2):e33168. doi: 10.2196/33168 (PMC8857691; doi:10.2196/33168)
Supplement: Multimedia Appendix 1 [file mhealth_v10i2e33168_app1.docx]

**Multimedia Appendix 1: Supplementary tables and figures**

**Figure S1. PRISMA Flow chart**

**Table S1. Search terms**

| **English terms** | **Danish terms** |
| --- | --- |
| Blood pressure, Hypertension | Blodtryk, Forhøjet blodtryk |
| Osteoarthritis, arthritis | Artrose, Slidgigt |
| Diabetes, type 2 diabetes | Diabetes |
| Chronic obstructive pulmonary disease, COPD | Kronisk obstruktiv lungesygdom, KOL |
| Depression, anxiety | Depression, Angst |
| Heart failure, ischemic heart disease, heart disease | Hjertesvigt, Iskæmisk hjertesygdom, hjertesygdom |
| Multimorbidity | Multisygdo |

**Table S2. Background characteristics of the Apps**

| **Condition and App name** | **Rating (0-5)** | | **Number of ratings** | | **Number of downloads** | | **Price** | **Language** | **Category** | **Store** | **App developer(s)** | **Version number** | **Last update** | **Size MB** | **Free of Advertisements** | **User ID required** | **Privacy policy** |
| --- | --- | --- | --- | --- | --- | --- | --- | --- | --- | --- | --- | --- | --- | --- | --- | --- | --- |
| ***Heart*** ***conditions*** |  | | | | | | | | | | | | | | | | |
| **Heart Failure Manager** | 5.0 | N/A | 2 | N/A | | N/A | Free | English | Medical | Apple | At Point of Care, LL | 10.6.0 | 28/05/20 | 23.9 | Yes | Yes | Yes |
| **Cardiogram: Heart Rate Monitor** | 4.3 | 4.3 | 4.800+ | 2.870 | | 1.000.000+ | In-app purchases | English | Health & Fitness | Apple & Google | Cardiogram Inc. | 3.2.1 | 20/11/20 | 61.1 | Yes | Yes | Yes |
| **Repair Your Heart Naturally** | N/A | 4.4 | N/A | 13 | | 1.000+ | Free | English | Health & Fitness | Google | IMAPPZ | 1.4 | 02/10/20 | 8.4 | No | No | No |
| **CardioVisual: Heart Health Built by Cardiologists** | 4.4 | 3.4 | 161 | 909 | | 100.000+ | In-app purchases | English | Medical | Apple & Google | MedicalVisual, Inc | 5.5.18 | 24/12/20 | 12 | Yes | Yes | Yes |
| **Instant Heart Rate: HR Monitor** | 4.9 | 4.0 | 153.000+ | 308.085 | | 10.000.000+ | In-app purchases | English | Health & Fitness | Apple & Google | Azumio Inc. | 5.342 | 18/11/20 | 247 | No | Yes | Yes |
| **Cardiio: Heart Rate Monitor** | 4.7 | N/A | 39.400+ | N/A | | N/A | In-app purchases | English (+13) | Health & Fitness | Apple | Cardiio, Inc. | 4.0.6 | 10/02/20 | 28.8 | Yes | No | Yes |
| ***Type 2 Diabetes*** | | | | | | | | | | | | | | | | | |
| **Jade Insulin Dose Calculator** | N/A | 3.2 | N/A | 75 | | 10.000+ | In-app purchases | English (+8) | Medical | Google | Jade Diabetes | 2.5.1 | 30/01/20 | 5.7 | Yes | Yes | Yes |
| **mySugr - Diabetes Tracker Log** | 4.7 | 4.6 | 8.100+ | 49.282 | | 1.000.000+ | In-app purchases | English (+22) | Medical | Apple & Google | mySugr GmbH | 3.86.1 | 19/12/20 | 85 | No | Yes | Yes |
| **Diabetes Tracker - Diabeto Log** | 4.6 | N/A | 517 | N/A | | N/A | In-app purchases | English (+7) | Medical | Apple | CUBESOFT SARL | 2020.28 | 14/12/20 | 62.1 | No | No | Yes |
| **Doctor Sugar - Diabetes Type 2** | 4.7 | N/A | 65 | N/A | | N/A | In-app purchases | English | Medical | Apple | Agnieszka Lisowska | 7.0 | 16/10/19 | 28.2 | Yes | No | Yes |
| **Diabetes 101** | 5.0 | N/A | 1 | N/A | | N/A | Free | English | Medical | Apple | Felecia Wood | not available | not available | 310.9 | Yes | No | No |
| **Diabetes Forum** | 4.4 | 4.1 | 87 | 872 | | 50.000+ | Free | English (+26) | Health & Fitness | Apple & Google | Diabetes Digital Media Ltd | 5.4.65 | 27/09/17 | 119.7 | Yes | Yes | Yes |
| **SocialDiabetes. Take control of your diabetes** | 4.4 | 4.2 | 41 | 3.467 | | 100.000+ | In-app purchases | English | Medical | Apple & Google | SocialDiabetes | 4.17.36 | 28/12/20 | 114 | Yes | Yes | Yes |
| **Glycemic Index Load Net Carbs** | 4.5 | 4.4 | 1.700+ | 11.890 | | 500.000+ | In-app purchases | English (+14) | Medical | Apple & Google | Rafal Platek | 3.6.1 | 05/11/20 | 9.8 | No | No | Yes |
| **Glucose Buddy Diabetes Tracker** | 4.8 | 4.0 | 18.400+ | 14.481 | | 100.000+ | In-app purchases | English (+30) | Medical | Apple & Google | Azumio Inc. | 5.274 | 02/10/20 | 236 | No | Yes | Yes |
| **Mumoactive Diabetes** | 4.6 | N/A | 10 | N/A | | N/A | Free | English | Health & Fitness | Apple | Strategic Specific Ltd. | 3.3 | 13/10/17 | 36.7 | Yes | Yes | Yes |
| ***Osteoarthritis*** | | | | | | | | | | | | | | | | | |
| **Jointfully Osteoarthritis** | 4.3 | 4.4 | 8 | 30 | | 1.000+ | Free | English (+1) | Health & Fitness | Apple & Google | Microhealth LLC | 1.0.15 | 09/01/20 | 33.1 | Yes | Yes | Yes |
| **Osteoarthritis Joint Pain Treatment Home Remedies** | N/A | 4.6 | N/A | 119 | | 10.000+ | In-app purchases | English | Health & Fitness | Google | Kaveri Tyagi | 2.0 | 25/11/20 | 6.5 | No | No | Yes |
| **ArthritisPower** | 3.8 | 3.7 | 13 | 53 | | 5.000+ | Free | English | Medical | Apple & Google | Global Healthy Living Foundation, Inc. | 2.7.2 | 09/10/20 | 42.4 | Yes | Yes | Yes |
| **Workout for Arthritis** | N/A | 3.0 | N/A | 59 | | 5.000+ | Free | English | Health & Fitness | Google | applogy | 4.0.3 | 12/08/18 | 4.8 | No | No | No |
| **Natural Remedies For Osteoarthritis** | N/A | // | N/A | 0 | | 100+ | Free | English | Health & Fitness | Google | TON GIAT PENG | 1.0 | 20/02/19 | 13 | No | No | Yes |
| **Osteoarthritis Disease** | N/A | // | N/A | 0 | | 500+ | Free | English | Health & Fitness | Google | bedieman | 1.0.0 | 28/11/17 | 6.1 | No | No | Yes |
| **Knee Pain Relieving Exercises** | N/A | 4.1 | N/A | 164 | | 50.000+ | Free | English (+1) | Health & Fitness | Google | Dr.Kavin Khatri | 1.0.0 | 31/12/16 | 4.1 | Yes | No | No |
| **Osteoarthritis (Gs)** | N/A | // | N/A | // | | 100+ | Free | English | Health & Fitness | Google | MRBELI | 1.0 | 07/02/19 | 3.1 | Yes | No | Yes |
| **Qigong for Arthritis Relief** | 5.0 | 4.2 | 1 | 33 | | 1.000+ | In-app purchases | English | Health & Fitness | Apple & Google | YMAA Publication Center, Inc. | 1.0.3 | 30/08/19 | 42.6 | Yes | No | Yes |
| ***Chronic obstructive pulmonary disease (COPD)*** |  | | | | | | | | | | | | | | | | |
| **COPD Pocket Consultant Guide** | 5.0 | 3.6 | 7 | 14 | | 1.000+ | Free | English | Medical | Apple & Google | COPD FOUNDATION, INC. | 3.0.1 | 17/10/19 | 43.1 | Yes | No | Yes |
| **Plan-it Med** | 5.0 | // | 5 | 1 | | 100+ | Free | English | Medical | Apple & Google | Play-it Health, LLC | 3.12.28 | 06/08/20 | 41.6 | Yes | Yes | Yes |
| **COPD GOLD** | N/A | 5.0 | N/A | 8 | | 1.000 + | Free | English | Medical | Google | Sebastian Sleiman | 1.2 | 20/09/18 | 2.4 | No | No | No |
| **Cliexa-COPD** | 5.0 | // | 2 | 0 | | 100+ | Free | English | Medical | Apple & Google | Collaborative Network 4 Clinical Exellence, LLC | 1.6 | 20/06/20 | 17.9 | Yes | Yes | Yes |
| **Hailie - Asthma & COPD Companion** | 3.7 | 4.0 | 7 | 12 | | 1.000+ | Free | English | Medical | Apple & Google | Adherium (NZ) Limited | 6.6.1.14 | 06/10/20 | 36 | Yes | Yes | Yes |
| **SaniQ Asthma** | 3.4 | 3.9 | 7 | 151 | | 5.000+ | In-app purchases | English | Medical | Apple & Google | Qurasoft GmbH | 3.9.5 | 25/09/20 | 57 | No | Yes | Yes |
| **Breath Ball: The Stress Relief Breathing Exercise.** | 4.8 | 4.7 | 73 | 1.705 | | 100.000+ | In-app purchases | English | Medical | Apple & Google | Fun Driven | 5.3.7 | 27/10/20 | 49 | Yes | No | Yes |
| **Universal Breathing - Pranayama Lite** | 3.4 | 4.5 | 24 | 1.103 | | 100.000+ | Free | English | Health & Fitness | Apple & Google | Saagara LLC | 2.10.1 | 07/08/17 | 90.6 | No | No | No |
| **COPD Manager** | 5.0 | N/A | 1 | N/A | | N/A | Free | English | Medical | Apple | At Point of Care, LLC | 10.6.1 | 10/09/20 | 31.5 | Yes | Yes | Yes |
| **EXi - Exercise Prescription** | 4.6 | 4.0 | 13 | 7 | | 1.000+ | Free | English | Medical | Apple & Google | IPrescribe Exercise Digital LTD | 2.2.1 | 26/10/20 | 138.1 | Yes | Yes | Yes |
| ***Hypertension*** | | | | | | | | | | | | | | | | | |
| **SmartBP - Smart Blood Pressure** | 4.3 | 3.5 | 11.100+ | 2.286 | | 500.000+ | In-app purchases | English (+17) | Medical | Apple & Google | Evolve Medical Systems, LLC | 6.1.1 | 03/12/20 | 176.2 | No | No | Yes |
| **Blood Pressure Tracker+** | 4.7 | N/A | 5.000+ | N/A | | N/A | In-app purchases | English (+6) | Medical | Apple | Adappt LLC | 2.8.8 | 29/12/20 | 36 | Yes | No | No |
| **Cora Health** | 4.4 | N/A | 146 | N/A | | N/A | In-app purchases | English (+9) | Medical | Apple | Swiftware OU | 1.6.4 | 27/10/20 | 118.9 | Yes | Yes | Yes |
| **Blood Pressure Companion** | 4.4 | N/A | 1.100+ | N/A | | N/A | Free | English (+9) | Medical | Apple | 倩 赵 | 7.2 | 09/04/20 | 21.5 | No | No | Yes |
| **Blood Pressure** | 4.6 | 4.2 | 114 | 45.863 | | 10.000.000+ | In-app purchases | English (+11) | Medical | Apple & Google | Klimaszewski Szymon | 6.1.3 | 11/12/20 | 14 | No | No | Yes |
| **Blood Pressure (BP) Diary** | 5.0 | 3.2 | 1 | 5.118 | | 1.000.000+ | In-app purchases | English (+12) | Medical | Apple & Google | openit Inc | 4.1.9 | 09/11/20 | 10 | No | Yes | Yes |
| **Welltory: EKG Heart Rate Monitor & HRV Stress Test** | 4.5 | 4.0 | 7.500+ | 10.576 | | 500.000+ | In-app purchases | English (+1) | Health & Fitness | Apple & Google | Welltory | 3.2.1 | 26/12/20 | 65 | No | Yes | Yes |
| **Blood Pressure Monitor** | 4.6 | N/A | 1.700+ | N/A | | N/A | In-app purchases | English (+6) | Medical | Apple | Taconic System LLC | 3.9.2 | 01/12/20 | 51.6 | No | Yes | Yes |
| **Blood Pressure Checker Diary -BP Info - BP Tracker** | N/A | 4.0 | N/A | 2.860 | | 500.000+ | Free | English | Medical | Google | Medical Health Comics | 4.0 | 31/08/20 | 4.6 | No | No | Yes |
| **Blood pressure app ++** | 4.9 | N/A | 841 | N/A | | N/A | In-app purchases | English | Health & Fitness | Apple | Touchberry Inc | 1.2.0 | 08/11/20 | 55.5 | Yes | No | Yes |
| ***Depression*** | | | | | | | | | | | | | | | | | |
| **TalkLife: Depression & Anxiety** | 4.5 | 4.2 | 2.800+ | 31.355 | | 1.000.000+ | In-app purchases | English | Health & Fitness | Apple & Google | TalkLife Limited | 6.3.74 | 27/12/20 | 246.1 | No | Yes | Yes |
| **Replika - My AI Friend** | 4.6 | 4.1 | 126.200+ | 305.613 | | 5.000.000+ | In-app purchases | English | Health & Fitness | Apple & Google | Luka, Inc. | 6.0.11 | 03/01/21 | 64 | Yes | Yes | Yes |
| **Motivation - Daily quotes** | 4.8 | 4.8 | 398.900+ | 19.484 | | 1.000.000+ | In-app purchases | English (+1) | Health & Fitness | Apple & Google | Monkey Taps | 2.8.5 | 31/12/20 | 73 | No | No | Yes |
| **Simple Habit Sleep, Meditation** | 4.8 | 4.7 | 70.800+ | 39.922 | | 1.000.000+ | In-app purchases | English | Health & Fitness | Apple & Google | Simple Habit, Inc. | 5.9.1 | 23/12/20 | 194.2 | Yes | Yes | Yes |
| **Wysa: Mental Health Support** | 4.8 | 4.8 | 4.200+ | 70.588 | | 1.000.000+ | In-app purchases | English | Health & Fitness | Apple & Google | Touchkin eServices Private Limited | 5.7.6 | 18/12/20 | 72.4 | Yes | No | Yes |
| **Youper: Self Care Therapy** | 4.9 | N/A | 14.200+ | N/A | | N/A | In-app purchases | English | Medical | Apple | YOUPER, INC. | 9.00.001 | 18/12/20 | 130.9 | Yes | Yes | Yes |
| **Sanvello: Anxiety & Depression** | 4.8 | 4.5 | 13.200+ | 19.403 | | 1.000.000+ | In-app purchases | English | Medical | Apple & Google | Sanvello Health Inc. | 8.20.0 | 21/12/20 | 129.5 | Yes | Yes | Yes |
| **MindDoc: Depression & Anxiety** | 4.7 | 4.5 | 27.100+ | 35.198 | | 1.000.000+ | In-app purchases | English (+1) | Medical | Apple & Google | MindDoc Health GmbH | 4.2.2 | 15/12/20 | 20.4 | Yes | Yes | Yes |
| **InnerHour Self-Care Therapy** | 4.0 | 4.6 | 23 | 6.793 | | 500.000+ | In-app purchases | English | Health & Fitness | Apple & Google | Mindcrescent Wellness Ventures Private Limited | 3.1 | 29/12/20 | 82.2 | Yes | Yes | Yes |
| **Remente: Self Help & Wellbeing** | 4.6 | 4.3 | 1.700+ | 10.433 | | 1.000.000+ | In-app purchases | English (+4) | Health & Fitness | Apple & Google | Remente AB | 1.3 | 29/12/20 | 79.7 | Yes | Yes | Yes |
| ***Multimorbidity*** | | | | | | | | | | | | | | | | | |
| **Withings Health Mate** | 4.6 | 4.2 | 150.900+ | 74.820 | | 1.000.000+ | Free | English (+10) | Health & Fitness | Apple & Google | Withings | 5.2.2 | 09/11/20 | 260.8 | Yes | Yes | Yes |
| **One Drop: Transform Your Life** | 4.4 | 4.0 | 16.900+ | 2.705 | | 1.000.000+ | In-app purchases | English (+10) | Health & Fitness | Apple & Google | Informed Data Systems, Inc | 6.6.0 | 18/12/20 | 277.7 | Yes | Yes | Yes |
| **CareClinic • Tracker, Reminder** | 4.8 | 3.5 | 798 | 264 | | 10.000+ | In-app purchases | English (+3) | Medical | Apple & Google | Tandem Loop Inc. | 2.15 | 03/12/20 | 53.6 | Yes | Yes | Yes |
| **Pill Reminder & Medication Tracker - MyTherapy** | 4.8 | 4.7 | 1.200+ | 86.972 | | 1.000.000+ | Free | English (+24) | Medical | Apple & Google | MyTherapy 💊⏰ | 3.79.1 | 04/12/20 | *247 | Yes | Yes | Yes |
| **Medisafe Medication Management** | 4.7 | 4.6 | 46.000+ | 209.586 | | 1.000.000+ | In-app purchases | English (+15) | Medical | Apple & Google | MediSafe Inc. | 6.7.41 | 31/12/20 | 173.5 | Yes | Yes | Yes |

**Table S3. Mobile App Rating Scale (MARS)**

|  | **Section A:**  **Engagement** | | | | | | **Section B:**  **Functionality** | | | | **Section C: Aesthetics** | | | **Section D:**  **Information** | | | | | | |  |
| --- | --- | --- | --- | --- | --- | --- | --- | --- | --- | --- | --- | --- | --- | --- | --- | --- | --- | --- | --- | --- | --- |
| **App Name and conditions** | **1. Entertainment** | **2. Interest** | **3. Customization** | **4. Interactivity** | **5. Target Group** | **6. Performance** | | **7. Ease of Use** | **8. Navigation** | **9. Gestural Design** | **10. Layout** | **11.Graphics** | **12. Visual Appeal** | **13. Accuracy** | **14. Goals*** | **15. Quality of info** | **16.Quantity of info** | **17. Visual info*** | **18. Credibility** | **19. Evidence Base*** | **App Quality Mean Overall Score** |
| ***Heart conditions*** |  | | | | | | | | | | | | | | | | | | | | |
| **Heart Failure Manager** | 4 | 4 | 4 | 4 | 4 | 3 | | 3 | 4 | 4 | 4 | 4 | 4 | 4 | 3 | 4 | 4 | 4 | 3 | N/A | 3,8 |
| **Cardiogram: Heart Rate Monitor** | 4 | 4 | 4 | 4 | 4 | 3 | | 4 | 4 | 4 | 4 | 4 | 4 | 4 | 4 | 4 | 4 | 4 | 3 | 3 | 3,9 |
| **Repair Your Heart Naturally** | 2 | 3 | 2 | 1 | 3 | 3 | | 4 | 4 | 2 | 3 | 2 | 2 | 4 | 2 | 4 | 3 | 2 | 2 | N/A | 2,7 |
| **CardioVisual: Heart Health Built by Cardiologists** | 3 | 3 | 2 | 2 | 4 | 3 | | 4 | 4 | 3 | 4 | 4 | 3 | 4 | 3 | 4 | 4 | 4 | 2 | N/A | 3,4 |
| **Instant Heart Rate: HR Monitor** | 4 | 4 | 3 | 3 | 4 | 4 | | 4 | 4 | 4 | 4 | 3 | 4 | 4 | 3 | 3 | 3 | 3 | 3 | 2 | 3,6 |
| **Cardiio: Heart Rate Monitor** | 4 | 4 | 3 | 3 | 3 | 4 | | 3 | 4 | 4 | 4 | 4 | 4 | 4 | 4 | 3 | 4 | 4 | 2 | 2 | 3,6 |
| ***Type 2 diabetes*** | | | | | | | | | | | | | | | | | | | | | |
| **Jade Insulin Dose Calculator** | 4 | 4 | 4 | 4 | 5 | 4 | | 3 | 4 | 3 | 4 | 3 | 3 | 5 | 4 | 4 | 4 | 3 | 3 | N/A | 3,7 |
| **mySugr - Diabetes Tracker Log** | 4 | 4 | 4 | 4 | 4 | 4 | | 4 | 4 | 4 | 4 | 3 | 4 | 4 | 4 | 4 | 4 | 3 | 3 | 4 | 3,8 |
| **Diabetes Tracker - Diabeto Log** | 3 | 4 | 4 | 4 | 4 | 4 | | 4 | 4 | 3 | 4 | 3 | 3 | 4 | 4 | 4 | 4 | 4 | 3 | N/A | 3,7 |
| **Doctor Sugar - Diabetes Type 2** | 2 | 2 | 2 | 3 | 4 | 4 | | 4 | 4 | 4 | 4 | 2 | 2 | 4 | 4 | 3 | 3 | 4 | 2 | N/A | 3,2 |
| **Diabetes 101** | 3 | 3 | 2 | 2 | 3 | 3 | | 3 | 4 | 3 | 3 | 3 | 2 | 2 | 2 | 4 | 2 | 3 | 4 | N/A | 2,8 |
| **Diabetes Forum** | 2 | 2 | 3 | 3 | 3 | 4 | | 3 | 4 | 4 | 3 | 3 | 3 | 4 | 3 | 3 | 3 | N/A | 3 | N/A | 3,1 |
| **SocialDiabetes. Take control of your diabetes** | 3 | 3 | 4 | 4 | 5 | 4 | | 4 | 4 | 4 | 4 | 4 | 4 | 4 | 3 | 4 | 3 | 4 | 3 | 3 | 3,8 |
| **Glycemic Index Load Net Carbs** | 3 | 3 | 4 | 3 | 4 | 4 | | 4 | 3 | 4 | 4 | 4 | 4 | 4 | 4 | 4 | 3 | 4 | 3 | N/A | 3,7 |
| **Glucose Buddy Diabetes Tracker** | 4 | 4 | 4 | 4 | 5 | 4 | | 3 | 4 | 4 | 4 | 4 | 4 | 5 | 4 | 4 | 4 | 4 | 3 | 4 | 4,0 |
| **Mumoactive Diabetes** | 2 | 3 | 2 | 3 | 3 | 4 | | 4 | 3 | 4 | 3 | 4 | 3 | 4 | 3 | 2 | 2 | 4 | 3 | N/A | 3,2 |
| ***Osteoarthritis*** |  | | | | | | | | | | | | | | | | | | | | |
| **Jointfully Osteoarthritis** | 4 | 4 | 3 | 4 | 4 | 5 | | 4 | 4 | 5 | 4 | 4 | 5 | 5 | 4 | 4 | 4 | 4 | 3 | N/A | 4,2 |
| **Osteoarthritis Joint Pain Treatment Home Remedies** | 3 | 3 | 1 | 1 | 4 | 4 | | 4 | 4 | 4 | 4 | 3 | 3 | 3 | 2 | 4 | 4 | 3 | 3 | N/A | 3,2 |
| **ArthritisPower** | 3 | 4 | 4 | 3 | 3 | 2 | | 3 | 3 | 2 | 3 | 3 | 3 | 4 | 4 | 4 | 4 | 3 | 4 | 3 | 3,2 |
| **Workout for Arthritis** | 2 | 2 | 2 | 2 | 4 | 4 | | 4 | 4 | 4 | 3 | 3 | 3 | 4 | 3 | 4 | 2 | 3 | 2 | N/A | 3,1 |
| **Natural Remedies For Osteoarthritis** | 2 | 2 | 1 | 1 | 3 | 4 | | 5 | 4 | 3 | 4 | 2 | 2 | 3 | 2 | 3 | 3 | N/A | 3 | N/A | 2,8 |
| **Osteoarthritis Disease** | 2 | 2 | 1 | 1 | 4 | 4 | | 4 | 3 | 2 | 2 | 2 | 2 | 3 | 2 | 3 | 3 | N/A | 2 | N/A | 2,5 |
| **Knee Pain Relieving Exercises** | 2 | 2 | 1 | 1 | 3 | 3 | | 4 | 3 | 2 | 3 | 2 | 2 | 4 | 3 | 4 | 3 | 3 | 2 | 2 | 2,5 |
| **Osteoarthritis** | 2 | 2 | 1 | 1 | 4 | 4 | | 4 | 4 | 3 | 4 | 2 | 2 | 3 | 2 | 4 | 3 | N/A | 2 | N/A | 2,8 |
| **Qigong for Arthritis Relief** | 2 | 2 | 1 | 1 | 3 | 4 | | 3 | 4 | 3 | 3 | 3 | 2 | 4 | 2 | 3 | 2 | 4 | 3 | N/A | 2,7 |
| ***Chronic obstructive pulmonary disease (COPD)*** |  | | | | | | | | | | | | | | | | | | | | |
| **COPD Pocket Consultant Guide** | 2 | 3 | 2 | 2 | 4 | 3 | | 3 | 4 | 3 | 3 | 3 | 3 | 3 | 3 | 5 | 4 | 3 | 3 | 3 | 3,1 |
| **Plan-it Med** | 4 | 3 | 3 | 4 | 3 | 4 | | 4 | 4 | 4 | 4 | 3 | 3 | 4 | 3 | 3 | 4 | 4 | 3 | N/A | 3,6 |
| **COPD GOLD** | 2 | 2 | 1 | 1 | 4 | 4 | | 4 | 3 | 2 | 3 | 2 | 2 | 3 | 2 | 4 | 2 | N/A | 2 | N/A | 2,5 |
| **Cliexa-COPD** | 3 | 3 | 3 | 3 | 4 | 4 | | 4 | 4 | 3 | 4 | 3 | 3 | 2 | 3 | 4 | 3 | 4 | 3 | N/A | 3,4 |
| **Hailie - Asthma & COPD Companion** | 3 | 3 | 3 | 2 | 4 | 3 | | 3 | 4 | 3 | 3 | 3 | 3 | 4 | 3 | 4 | 3 | 3 | 3 | 3 | 3,1 |
| **SaniQ Asthma** | 3 | 4 | 3 | 4 | 4 | 4 | | 3 | 4 | 3 | 4 | 3 | 4 | 4 | 4 | 4 | 4 | 4 | 3 | 3 | 3,6 |
| **Breath Ball: The Stress Relief Breathing Exercise.** | 3 | 3 | 3 | 2 | 3 | 4 | | 4 | 4 | 4 | 4 | 3 | 3 | 5 | 3 | 4 | 3 | 3 | 3 | N/A | 3,4 |
| **Universal Breathing - Pranayama Lite** | 3 | 2 | 2 | 1 | 2 | 4 | | 2 | 4 | 3 | 4 | 3 | 3 | 4 | 2 | 2 | 2 | N/A | 3 | 3 | 2,8 |
| **COPD Manager** | 4 | 4 | 4 | 3 | 4 | 3 | | 4 | 4 | 4 | 4 | 4 | 4 | 4 | 4 | 4 | 4 | 4 | 3 | 2 | 3,8 |
| **EXi - Exercise Prescription** | 3 | 4 | 4 | 3 | 4 | 3 | | 4 | 3 | 4 | 4 | 4 | 3 | 4 | 4 | 4 | 3 | 4 | 3 | N/A | 3,6 |
| ***Hypertension*** |  | | | | | | | | | | | | | | | | | | | | |
| **SmartBP - Smart Blood Pressure** | 3 | 4 | 3 | 3 | 4 | 4 | | 4 | 4 | 4 | 4 | 4 | 3 | 4 | 4 | 4 | 3 | 4 | 3 | 3 | 3,7 |
| **Blood Pressure Tracker+** | 4 | 4 | 3 | 4 | 4 | 4 | | 3 | 4 | 4 | 4 | 4 | 4 | 3 | 3 | 4 | 4 | 4 | 2 | N/A | 3,7 |
| **Cora Health** | 4 | 3 | 4 | 3 | 4 | 4 | | 4 | 4 | 3 | 4 | 4 | 4 | 4 | 4 | 4 | 3 | 4 | 3 | 3 | 3,7 |
| **Blood Pressure Companion** | 2 | 3 | 3 | 4 | 3 | 3 | | 4 | 3 | 3 | 3 | 3 | 3 | 4 | 3 | 3 | 2 | 3 | 3 | 3 | 3,1 |
| **Blood Pressure** | 4 | 4 | 4 | 3 | 4 | 3 | | 4 | 4 | 3 | 3 | 3 | 4 | 4 | 4 | 4 | 3 | 4 | 3 | 3 | 3,6 |
| **Blood Pressure (BP) Diary** | 3 | 4 | 4 | 4 | 4 | 3 | | 3 | 4 | 4 | 4 | 4 | 4 | 4 | 4 | 4 | 3 | 4 | 3 | N/A | 3,7 |
| **Welltory: EKG Heart Rate Monitor & HRV Stress Test** | 4 | 4 | 4 | 4 | 4 | 4 | | 4 | 4 | 4 | 4 | 4 | 5 | 4 | 4 | 4 | 4 | 4 | 3 | 3 | 4,0 |
| **Blood Pressure Monitor** | 3 | 3 | 3 | 3 | 3 | 4 | | 4 | 4 | 4 | 4 | 4 | 3 | 4 | 3 | 4 | 3 | 4 | 3 | N/A | 3,5 |
| **Blood Pressure Checker Diary -BP Info - BP Tracker** | 2 | 3 | 2 | 2 | 4 | 4 | | 4 | 4 | 3 | 4 | 2 | 2 | 2 | 2 | 3 | 3 | 3 | 2 | N/A | 2,9 |
| **Blood pressure app ++** | 3 | 4 | 3 | 3 | 4 | 4 | | 4 | 4 | 4 | 4 | 3 | 3 | 4 | 3 | 4 | 3 | 3 | 2 | N/A | 3,5 |
| ***Depression*** |  | | | | | | | | | | | | | | | | | | | | |
| **TalkLife: Depression & Anxiety** | 4 | 4 | 4 | 3 | 4 | 4 | | 4 | 4 | 4 | 4 | 4 | 4 | 4 | 3 | 3 | 2 | 4 | 3 | 3 | 3,7 |
| **Replika - My AI Friend** | 5 | 4 | 4 | 3 | 3 | 4 | | 4 | 4 | 4 | 4 | 3 | 5 | 4 | 3 | 2 | 2 | 4 | 3 | 3 | 3,7 |
| **Motivation - Daily quotes** | 3 | 4 | 3 | 3 | 3 | 3 | | 4 | 4 | 4 | 4 | 4 | 4 | 4 | 3 | 2 | 3 | 4 | 3 | 2 | 3,5 |
| **Simple Habit Sleep, Meditation** | 4 | 4 | 4 | 3 | 3 | 5 | | 4 | 4 | 4 | 4 | 4 | 5 | 4 | 3 | 3 | 3 | 4 | 3 | 2 | 3,8 |
| **Wysa: Mental Health Support** | 5 | 4 | 4 | 4 | 4 | 5 | | 4 | 4 | 4 | 4 | 5 | 5 | 4 | 3 | 4 | 5 | 5 | 2 | 3 | 4,2 |
| **Youper: Self Care Therapy** | 4 | 4 | 4 | 5 | 4 | 4 | | 4 | 4 | 4 | 4 | 4 | 5 | 4 | 4 | 4 | 5 | 3 | 3 | 3 | 4,1 |
| **Sanvello: Anxiety & Depression** | 5 | 5 | 4 | 4 | 4 | 4 | | 4 | 4 | 5 | 4 | 5 | 5 | 4 | 4 | 4 | 4 | 4 | 3 | 4 | 4,3 |
| **MindDoc: Depression & Anxiety** | 4 | 3 | 4 | 3 | 4 | 4 | | 4 | 3 | 4 | 4 | 4 | 4 | 4 | 3 | 4 | 4 | 4 | 3 | 3 | 3,7 |
| **InnerHour: Self-Care Therapy** | 4 | 5 | 4 | 4 | 4 | 4 | | 4 | 4 | 4 | 4 | 4 | 5 | 4 | 4 | 4 | 4 | 4 | 3 | 2 | 4,0 |
| **Remente: Self Help & Wellbeing** | 4 | 4 | 4 | 4 | 4 | 4 | | 3 | 4 | 4 | 4 | 4 | 4 | 4 | 4 | 4 | 4 | 4 | 3 | 3 | 3,9 |
| ***Multimorbidity*** | | | | | | | | | | | | | | | | | | | | | |
| **Withings Health Mate** | 4 | 4 | 4 | 4 | 4 | 4 | | 3 | 4 | 4 | 4 | 3 | 4 | 4 | 4 | 4 | 4 | 3 | 3 | 5 | 3,8 |
| **One Drop: Transform Your Life** | 4 | 4 | 3 | 3 | 4 | 4 | | 4 | 4 | 3 | 4 | 3 | 4 | 4 | 4 | 4 | 4 | 4 | 3 | 4 | 3,7 |
| **CareClinic • Tracker, Reminder** | 3 | 3 | 4 | 4 | 4 | 3 | | 3 | 3 | 4 | 4 | 4 | 3 | 4 | 3 | 3 | 3 | 3 | 3 | N/A | 3,4 |
| **Pill Reminder & Medication Tracker - MyTherapy** | 3 | 4 | 4 | 4 | 3 | 4 | | 4 | 3 | 4 | 4 | 3 | 3 | 5 | 4 | 4 | 4 | 4 | 3 | 3 | 3,6 |
| **Medisafe Medication Management** | 3 | 3 | 4 | 3 | 3 | 4 | | 4 | 4 | 3 | 4 | 4 | 3 | 4 | 3 | 4 | 3 | 4 | 3 | 5 | 3,6 |

**Table S4. Mobile App Rating Scale (MARS) subjective assessment.**

| **Condition and App name** | **1. Would you recommend this app to people who might benefit from it?** | | **2. How many times do you think you would use this app in the next 12 months if it was relevant to**  **you?** | | **3. Would you pay for this app?** | **4. What is your overall star rating of the app?** | **Mean Score:** |
| --- | --- | --- | --- | --- | --- | --- | --- |
| **Heart conditions** |  | |  | |  |  |  |
| **Heart Failure Manager** | 3 | | 4 | | 3 | 3 | 3,3 |
| **Cardiogram: Heart Rate Monitor** | 4 | | 4 | | 3 | 4 | 3,8 |
| **Repair Your Heart Naturally** | 2 | | 3 | | 1 | 2 | 2 |
| **CardioVisual: Heart Health Built by Cardiologists** | 4 | | 3 | | 1 | 3 | 2,8 |
| **Instant Heart Rate: HR Monitor** | 3 | | 4 | | 3 | 3 | 3,3 |
| **Cardiio: Heart Rate Monitor** | 3 | | 4 | | 3 | 4 | 3,5 |
| **Type 2 diabetes** |  | |  |  | | |  |
| **Jade Insulin Dose Calculator** | 3 | | 4 | | 3 | 4 | 3,5 |
| **mySugr - Diabetes Tracker Log** | 4 | | 4 | | 3 | 4 | 3,8 |
| **Diabetes Tracker - Diabeto Log** | 3 | | 4 | | 3 | 4 | 3,5 |
| **Doctor Sugar - Diabetes Type 2** | 3 | | 4 | | 1 | 3 | 2,8 |
| **Diabetes 101** | 3 | | 3 | | 1 | 2 | 2,3 |
| **Diabetes Forum** | 3 | | 3 | | 1 | 3 | 2,5 |
| **SocialDiabetes. Take control of your diabetes** | 4 | | 4 | | 3 | 4 | 3,8 |
| **Glycemic Index Load Net Carbs** | 3 | | 4 | | 3 | 4 | 3,5 |
| **Glucose Buddy Diabetes Tracker** | 4 | | 3 | | 4 | 4 | 3,8 |
| **Mumoactive Diabetes** | 3 | | 3 | | 3 | 3 | 3,0 |
| **Osteoarthritis** |  | | | | | | |
| **Jointfully Osteoarthritis** | 4 | 5 | | | 5 | 4 | 4,5 |
| **Osteoarthritis Joint Pain Treatment Home Remedies** | 3 | 3 | | | 1 | 3 | 2,5 |
| **ArthritisPower** | 4 | 5 | | | 3 | 4 | 4,0 |
| **Workout for Arthritis** | 2 | 3 | | | 1 | 2 | 2,0 |
| **Natural Remedies For Osteoarthritis** | 2 | 2 | | | 1 | 2 | 1,8 |
| **Osteoarthritis Disease** | 2 | 2 | | | 1 | 2 | 1,8 |
| **Knee Pain Relieving Exercises** | 2 | 3 | | | 1 | 3 | 2,3 |
| **Osteoarthritis (Gs)** | 2 | 2 | | | 1 | 2 | 1,8 |
| **Qigong for Arthritis Relief** | 2 | 2 | | | 1 | 2 | 1,8 |
| ***Chronic obstructive pulmonary disease (COPD)*** |  | | | | | | |
| **COPD Pocket Consultant Guide** | 2 | 3 | | | 2 | 3 | 2,5 |
| **Plan-it Med** | 3 | 4 | | | 3 | 4 | 3,5 |
| **COPD GOLD** | 2 | 3 | | | 1 | 2 | 2,0 |
| **Cliexa-COPD** | 3 | 3 | | | 1 | 3 | 2,5 |
| **Hailie - Asthma & COPD Companion** | 3 | 4 | | | 3 | 3 | 3,3 |
| **SaniQ Asthma** | 4 | 4 | | | 3 | 3 | 3,5 |
| **Breath Ball: The Stress Relief Breathing Exercise.** | 3 | 3 | | | 1 | 3 | 2,5 |
| **Universal Breathing - Pranayama Lite** | 2 | 2 | | | 1 | 3 | 2,0 |
| **COPD Manager** | 4 | 4 | | | 3 | 4 | 3,8 |
| **EXi - Exercise Prescription** | 4 | 4 | | | 3 | 3 | 3,5 |
| ***Hypertension*** |  | | | | | | |
| **SmartBP - Smart Blood Pressure** | 3 | 3 | | | 2 | 3 | 2,8 |
| **Blood Pressure Tracker+** | 3 | 4 | | | 3 | 4 | 3,5 |
| **Cora Health** | 4 | 4 | | | 3 | 4 | 3,8 |
| **Blood Pressure Companion** | 2 | 3 | | | 2 | 2 | 2,3 |
| **Blood Pressure** | 3 | 4 | | | 3 | 4 | 3,5 |
| **Blood Pressure (BP) Diary** | 3 | 4 | | | 3 | 4 | 3,5 |
| **Welltory: EKG Heart Rate Monitor & HRV Stress Test** | 3 | 4 | | | 5 | 4 | 4,0 |
| **Blood Pressure Monitor** | 3 | 3 | | | 2 | 3 | 2,8 |
| **Blood Pressure Checker Diary -BP Info - BP Tracker** | 2 | 2 | | | 1 | 2 | 1,8 |
| **Blood pressure app ++** | 3 | 4 | | | 3 | 3 | 3,3 |
| **Depression** |  | | | | | | |
| **TalkLife: Depression & Anxiety** | 3 | 3 | | | 3 | 4 | 3,3 |
| **Replika - My AI Friend** | 3 | 4 | | | 3 | 3 | 3,3 |
| **Motivation - Daily quotes** | 3 | 3 | | | 3 | 3 | 3,0 |
| **Simple Habit Sleep, Meditation** | 3 | 3 | | | 3 | 4 | 3,3 |
| **Wysa: Mental Health Support** | 4 | 4 | | | 3 | 3 | 3,5 |
| **Youper: Self Care Therapy** | 4 | 3 | | | 5 | 4 | 4,0 |
| **Sanvello: Anxiety & Depression** | 4 | 4 | | | 5 | 3 | 4,0 |
| **MindDoc: Depression & Anxiety** | 3 | 3 | | | 3 | 4 | 3,3 |
| **InnerHour: Self-Care Therapy** | 4 | 4 | | | 3 | 4 | 3,8 |
| **Remente: Self Help & Wellbeing** | 3 | 4 | | | 3 | 4 | 3,5 |
| **Multimorbidity** |  | | | | | | |
| **Withings Health Mate** | 3 | 4 | | | 3 | 4 | 3,5 |
| **One Drop: Transform Your Life** | 4 | 4 | | | 3 | 4 | 3,8 |
| **CareClinic • Tracker, Reminder** | 2 | 3 | | | 2 | 3 | 2,5 |
| **Pill Reminder & Medication Tracker - MyTherapy** | 4 | 4 | | | 3 | 4 | 3,8 |
| **Medisafe Medication Management** | 3 | 4 | | | 3 | 3 | 3,3 |

**Table S5. App Behaviour Change Scale (ABACUS) assessment.**

|  | **App Behavior Change Scale (ABACUS)** | | | | | | | | | | | | | | | | | | | | | | | | |
| --- | --- | --- | --- | --- | --- | --- | --- | --- | --- | --- | --- | --- | --- | --- | --- | --- | --- | --- | --- | --- | --- | --- | --- | --- | --- |
|  | **Section 1:**  **Knowledge and Information** | | | | | | **Section 2:**  **Goals and Planning** | | | | **Section 3:**  **Feedback and Monitoring** | | | | | | | | **Section 4:**  **Actions** | | | | | |  |
| **Condition and App name** | 1.1 Customize and personalize features | 1.2 Consistent with national guidelines or created with expertise | 1.3 Baseline information | 1.4 Instruction on how to perform the behavior | 1.5 Information about the consequences of continuing and/or discontinuing behavior | 2.2 Willingness for behavior change | | 2.2 Goal setting | 2.3 Review goals, update, and change when necessary | 3.1 Understand the difference between current action and future goals | | 3.2 Self-monitor behavior | 3.3 Share behaviors with others and/or allow for social comparison | 3.4 User feedback (in person or automatically) | 3.5 Export data | 3.6 Material or social reward or incentive | 3.7 General encouragement | 4.1 Reminders and/or prompts or cues for activity | | 4.2 Encourage positive habit formation | 4.3 Practice or rehearsal, in addition to daily activities | 4.4 Opportunity to plan for barriers | 4.5 Restructuring the physical or social environment | 4.6 Distraction or avoidance | **ABACUS overall score** |
| **Heart conditions** |  | | | | | | | | | | | | | | | | | | | | | | | | |
| **Heart Failure Manager** | Yes | Yes | Yes | No | Yes | No | | No | No | No | | Yes | Yes | No | Yes | Yes | No | Yes | | No | Yes | No | No | No | 10 |
| **Cardiogram: Heart Rate Monitor** | Yes | Yes | Yes | No | No | No | | No | No | No | | Yes | Yes | Yes | Yes | Yes | No | No | | Yes | Yes | No | No | No | 10 |
| **Repair Your Heart Naturally** | No | Yes | Yes | No | No | No | | No | No | No | | Yes | No | No | No | No | No | No | | No | Yes | No | No | No | 4 |
| **CardioVisual: Heart Health Built by Cardiologists** | Yes | Yes | No | No | No | No | | No | No | No | | Yes | No | No | No | No | No | No | | No | Yes | No | No | No | 4 |
| **Instant Heart Rate: HR Monitor** | Yes | Yes | Yes | No | No | No | | No | No | No | | Yes | No | No | No | No | No | No | | No | Yes | No | No | No | 5 |
| **Cardiio: Heart Rate Monitor** | Yes | Yes | Yes | Yes | No | No | | No | No | No | | Yes | Yes | Yes | Yes | Yes | No | Yes | | Yes | Yes | No | No | No | 12 |
| **Type 2 Diabetes** |  | | | | | | | | | | | | | | | | | | | | | | | | |
| **Jade Insulin Dose Calculator** | Yes | Yes | Yes | No | No | No | | Yes | Yes | Yes | | Yes | Yes | No | Yes | Yes | No | Yes | | No | Yes | No | No | No | 12 |
| **mySugr - Diabetes Tracker Log** | Yes | Yes | Yes | No | No | No | | Yes | Yes | Yes | | Yes | No | No | No | Yes | Yes | Yes | | Yes | Yes | No | No | No | 12 |
| **Diabetes Tracker - Diabeto Log** | Yes | Yes | Yes | No | No | No | | Yes | Yes | No | | Yes | No | No | Yes | No | No | Yes | | No | Yes | No | No | No | 9 |
| **Doctor Sugar - Diabetes Type 2** | No | Yes | No | No | No | No | | No | No | No | | Yes | No | No | Yes | No | No | Yes | | No | Yes | No | No | No | 5 |
| **Diabetes 101** | No | Yes | No | Yes | Yes | No | | No | No | No | | No | No | No | No | No | No | No | | No | No | No | No | No | 3 |
| **Diabetes Forum** | Yes | No | No | No | No | No | | No | No | No | | No | Yes | No | No | No | No | No | | No | No | No | No | No | 2 |
| **SocialDiabetes. Take control of your diabetes** | Yes | Yes | Yes | No | No | No | | Yes | Yes | Yes | | Yes | No | Yes | Yes | No | No | Yes | | No | Yes | No | No | No | 11 |
| **Glycemic Index Load Net Carbs** | Yes | Yes | Yes | No | No | No | | Yes | Yes | No | | Yes | No | No | No | No | Yes | Yes | | No | Yes | No | No | No | 9 |
| **Glucose Buddy Diabetes Tracker** | Yes | Yes | Yes | No | No | No | | No | No | No | | Yes | No | Yes | Yes | No | No | Yes | | No | Yes | No | No | No | 8 |
| **Mumoactive Diabetes** | Yes | Yes | No | No | No | No | | No | No | No | | Yes | No | No | Yes | No | No | Yes | | No | Yes | No | No | No | 6 |
| **Osteoarthritis** |  | | | | | | | | | | | | | | | | | | | | | | | | |
| **Jointfully Osteoarthritis** | Yes | Yes | Yes | Yes | No | No | | Yes | No | No | | Yes | No | Yes | No | No | No | Yes | | No | Yes | No | No | No | 7 |
| **Osteoarthritis Joint Pain Treatment Home Remedies** | No | Yes | No | Yes | No | No | | No | No | No | | No | No | No | No | No | No | No | | No | Yes | No | Yes | No | 6 |
| **ArthritisPower** | Yes | Yes | No | No | No | No | | No | No | Yes | | Yes | No | No | Yes | No | No | Yes | | No | Yes | No | No | No | 3 |
| **Workout for Arthritis** | No | Yes | No | Yes | Yes | No | | No | No | No | | No | No | No | No | No | No | No | | Yes | Yes | No | No | No | 5 |
| **Natural Remedies For Osteoarthritis** | No | No | No | No | No | No | | No | No | No | | No | No | No | No | No | No | No | | Yes | Yes | No | No | No | 6 |
| **Osteoarthritis Disease** | No | Yes | No | No | No | No | | No | No | No | | No | No | No | No | No | No | No | | No | Yes | No | No | No | 8 |
| **Knee Pain Relieving Exercises** | No | Yes | No | Yes | Yes | No | | No | No | No | | No | No | No | No | No | No | No | | No | Yes | No | Yes | No | 5 |
| **Osteoarthritis** | No | Yes | No | No | No | No | | No | No | No | | No | No | No | No | No | No | No | | No | Yes | No | No | No | 9 |
| **Qigong for Arthritis Relief** | No | No | No | Yes | No | No | | No | No | No | | No | No | No | No | No | No | No | | No | Yes | No | No | No | 13 |
| ***COPD: Chronic obstructive pulmonary disease (COPD)*** |  | | | | | | | | | | | | | | | | | | | | | | | | |
| **COPD Pocket Consultant Guide** | Yes | Yes | No | Yes | No | No | | No | No | No | | Yes | Yes | No | Yes | No | No | No | | No | Yes | No | No | No | 7 |
| **Plan-it Med** | Yes | Yes | Yes | No | No | No | | No | No | No | | Yes | No | No | No | No | No | Yes | | No | Yes | No | No | No | 6 |
| **COPD GOLD** | No | Yes | No | No | No | No | | No | No | No | | Yes | No | No | No | No | No | No | | No | Yes | No | No | No | 3 |
| **Cliexa-COPD** | Yes | Yes | Yes | No | No | No | | No | No | No | | Yes | No | No | No | No | No | No | | No | Yes | No | No | No | 5 |
| **Hailie - Asthma & COPD Companion** | Yes | Yes | No | No | No | No | | No | No | Yes | | Yes | Yes | No | No | No | No | No | | No | Yes | No | No | No | 6 |
| **SaniQ Asthma** | Yes | Yes | Yes | No | No | No | | No | No | No | | Yes | No | Yes | Yes | No | No | Yes | | No | Yes | No | No | No | 8 |
| **Breath Ball: The Stress Relief Breathing Exercise.** | Yes | Yes | No | Yes | No | No | | No | No | No | | No | No | No | No | No | No | No | | No | Yes | No | No | No | 4 |
| **Universal Breathing - Pranayama Lite** | Yes | Yes | No | Yes | No | No | | No | No | No | | Yes | No | No | No | No | No | No | | No | Yes | No | No | No | 5 |
| **COPD Manager** | Yes | Yes | Yes | No | Yes | No | | No | No | No | | Yes | No | No | No | Yes | No | Yes | | Yes | Yes | No | No | No | 9 |
| **EXi - Exercise Prescription** | Yes | Yes | Yes | No | No | No | | Yes | Yes | Yes | | Yes | Yes | Yes | Yes | Yes | No | Yes | | No | Yes | No | No | No | 14 |
| **Hypertension** |  | | | | | | | | | | | | | | | | | | | | | | | | |
| **SmartBP - Smart Blood Pressure** | Yes | Yes | Yes | No | No | No | | No | No | No | | Yes | No | No | Yes | No | No | Yes | | No | No | No | No | No | 6 |
| **Blood Pressure Tracker+** | Yes | Yes | Yes | No | No | No | | No | No | No | | Yes | No | No | Yes | No | No | Yes | | No | Yes | No | Yes | No | 8 |
| **Cora Health** | Yes | Yes | Yes | No | No | No | | Yes | Yes | Yes | | Yes | No | Yes | No | Yes | No | Yes | | Yes | Yes | No | No | No | 12 |
| **Blood Pressure Companion** | Yes | Yes | Yes | No | No | No | | Yes | Yes | Yes | | Yes | Yes | No | Yes | No | No | Yes | | No | No | No | No | No | 10 |
| **Blood Pressure** | Yes | Yes | Yes | No | No | No | | No | No | No | | Yes | No | Yes | Yes | No | No | Yes | | No | Yes | No | No | No | 8 |
| **Blood Pressure (BP) Diary** | Yes | Yes | Yes | No | No | No | | Yes | Yes | Yes | | Yes | No | No | Yes | No | No | Yes | | No | Yes | No | No | No | 10 |
| **Welltory: EKG Heart Rate Monitor & HRV Stress Test** | Yes | Yes | Yes | No | No | No | | No | No | Yes | | Yes | No | Yes | Yes | No | No | Yes | | No | Yes | No | Yes | No | 10 |
| **Blood Pressure Monitor** | Yes | Yes | Yes | No | No | No | | No | No | No | | Yes | No | No | Yes | No | No | Yes | | No | No | No | No | No | 6 |
| **Blood Pressure Checker Diary -BP Info - BP Tracker** | Yes | Yes | No | No | No | No | | No | No | Yes | | Yes | No | No | No | No | No | No | | No | Yes | No | No | No | 5 |
| **Blood pressure app ++** | Yes | Yes | No | No | No | No | | No | No | No | | Yes | No | No | No | No | No | No | | No | Yes | No | No | No | 4 |
| **Depression** |  | | | | | | | | | | | | | | | | | | | | | | | | |
| **TalkLife: Depression & Anxiety** | Yes | Yes | No | No | No | No | | No | No | No | | No | Yes | Yes | No | Yes | Yes | No | | No | Yes | No | No | No | 7 |
| **Replika - My AI Friend** | Yes | Yes | No | No | No | No | | No | No | No | | No | No | Yes | No | Yes | No | Yes | | No | Yes | No | No | No | 6 |
| **Motivation - Daily quotes** | Yes | No | No | No | No | No | | No | No | No | | No | No | No | Yes | No | Yes | Yes | | No | Yes | No | No | No | 5 |
| **Simple Habit Sleep, Meditation** | Yes | Yes | No | Yes | No | No | | No | No | No | | Yes | Yes | No | Yes | No | Yes | Yes | | Yes | Yes | No | No | No | 10 |
| **Wysa: Mental Health Support** | Yes | Yes | No | Yes | Yes | Yes | | No | No | No | | No | No | Yes | No | No | Yes | Yes | | Yes | Yes | No | No | No | 9 |
| **Youper: Self Care Therapy** | Yes | Yes | No | Yes | Yes | Yes | | Yes | Yes | No | | Yes | Yes | Yes | Yes | No | Yes | Yes | | Yes | Yes | No | No | No | 15 |
| **Sanvello: Anxiety & Depression** | Yes | Yes | Yes | Yes | Yes | No | | Yes | Yes | Yes | | Yes | Yes | Yes | Yes | Yes | Yes | Yes | | Yes | Yes | No | No | No | 18 |
| **MindDoc: Depression & Anxiety** | Yes | Yes | No | Yes | Yes | No | | No | No | No | | No | No | Yes | No | No | Yes | Yes | | Yes | Yes | No | No | No | 9 |
| **InnerHour: Self-Care Therapy** | Yes | Yes | No | Yes | Yes | No | | Yes | Yes | Yes | | Yes | No | Yes | No | No | Yes | Yes | | Yes | Yes | No | No | No | 13 |
| **Remente: Self Help & Wellbeing** | Yes | Yes | No | Yes | No | No | | Yes | Yes | Yes | | Yes | No | No | No | No | Yes | Yes | | Yes | Yes | No | No | No | 11 |
| **Multimorbidity** |  | | | | | | | | | | | | | | | | | | | | | | | | |
| **Withings Health Mate** | Yes | Yes | Yes | No | Yes | No | | Yes | Yes | Yes | | Yes | Yes | Yes | Yes | Yes | Yes | Yes | | Yes | Yes | No | No | No | 16 |
| **One Drop for Diabetes Health** | Yes | Yes | Yes | No | No | No | | Yes | Yes | Yes | | Yes | Yes | Yes | Yes | No | No | Yes | | Yes | Yes | Yes | No | No | 14 |
| **CareClinic • Tracker, Reminder** | Yes | Yes | Yes | Yes | No | No | | Yes | Yes | Yes | | Yes | No | Yes | No | No | No | Yes | | No | Yes | No | No | No | 11 |
| **Pill Reminder & Medication Tracker - MyTherapy** | Yes | Yes | Yes | No | No | No | | Yes | Yes | Yes | | Yes | Yes | Yes | Yes | No | No | Yes | | No | Yes | No | No | No | 12 |
| **Medisafe Medication Management** | Yes | Yes | Yes | No | No | No | | No | No | No | | Yes | No | Yes | Yes | No | No | Yes | | No | No | No | No | No | 7 |
